# Olive Oil Phenolics and Platelets—From Molecular Mechanisms to Human Studies

**DOI:** 10.31083/j.rcm2308255

**Published:** 2022-07-19

**Authors:** Maria Efthymia Katsa, Tzortzis Nomikos

**Affiliations:** ^1^Department of Nutrition and Dietetics, School of Health Sciences and Education, Harokopio University, 176 76 Athens, Greece

**Keywords:** platelets, Mediterranean Diet, olive oil, polyphenols, hydroxytyrosol, oleuropein, cardiovascular diseases, postprandial, dietary intervention study, animal models

## Abstract

Chronically activated, dysfunctional platelets mediate the progression of the majority of non-communicable diseases in a pleiotropic fashion. Antiplatelet therapy remains an attractive therapeutic means which however hasn’t reached the expected targets according to the promising preclinical studies. It is therefore obvious that the consumption of foods demonstrating antiplatelet activity may be a less drastic but on the other hand a more sustainable way of achieving daily antiplatelet therapy, either alone or in combination with antiplatelet drugs. Olive oil is probably the main cardioprotective component of the Mediterranean Diet according to the results of observational and dietary intervention studies. Among all phytochemicals of olive oil, its unique phenolics seems to be responsible for the majority of its cardioprotective properties. This review article aims to highlight the platelet modulating roles of olive oil polyphenols, trying to critically assess whether those properties could partially explain the cardioprotective role of olive oil. The cellular and animal studies clearly show that extra virging olive oil (EVOO) phenolics, mainly hydroxytyrosol, are able to inhibit the activation of platelets induced by several endogenous agonists and pathologies. However, the outcomes of the pre-clinical studies are difficult to be translated to humans mainly because the dosages and the chemical forms of the phenolics used to these studies are much higher and different to that found in human circulation. Despite the heterogeneity of the few human trials on the field so far, the results are promising showing that EVOO can exert antiplatelet actions in real, acute or long-term, trials and at least part of this antiplatelet action can be attributed to the phenolic content of EVOOs. Although we clearly need better, well-powered studies to give certain answers on this field the antiplatelet properties of olive oil phenolics is a promising, emerging mechanism which may explain some of the health properties of EVOO and the Mediterranean Diet.

## 1. Introduction 

The cardioprotective role of the Mediterranean Diet (MD) has long been established mainly due to the outcomes of large epidemiological studies showing that a high adherence to this prudent dietary model is inversely related to the risk of cardiovascular disease (CVD) hard end-points like myocardial infarction (MI), stroke, cardiovascular morbidity and mortality [1, 2]. Although the countries of the Mediterranean basin follow slightly different variations of the MD [3], the consumption of olive oil (OO), as the main dietary fat source, is a central, common trait of all types of the MD. It is therefore obvious that the biological properties of OO, mainly those related to the protection from CVDs, has been studied extensively till now [4]. The cardioprotective properties of OO have been initially attributed to its high content of monounsaturated fatty acids (MUFA), especially oleic acid. However, we now know that a small number of OO’s phytochemicals, especially polyphenols, show a plethora of biological properties which seem to be responsible for the health benefits of OO [5].

Platelets are physiological cellular effectors of haemostasis, innate immunity and tissue regeneration [6]. However, in the last two decades their implication in several mechanisms underlying chronic diseases has been highlighted [7, 8, 9]. A chronically activated or a dysfunctional platelet may initiate and propagate cellular mechanisms in the vascular bed which lead to atherosclerosis, atherothrombosis, vascular inflammation and unregulated haemostasis [10]. The antiplatelet pharmacology, although necessary for the primary and secondary prevention of CVDs, faces many challenges including bleeding complications and resistance of platelets to drug actions [11, 12]. Nutrition is a daily mild modulator of platelet actions and can either directly or indirectly affect platelet functionality. Dietary patterns, micronutrients and phytochemicals able to normalize the biological functions of platelets could be proven beneficial for the prevention of CVDs [13, 14]. The antiplatelet properties of diet is a rather ignored aspect of it, however, taking into account the crucial mediating role of platelets in many pathological mechanisms of CVDs, it can partially explain the cardioprotective properties of prudent dietary patterns such the MD pattern.

This review article aims to highlight the platelet modulating roles of OO polyphenols, trying to critically assess whether those properties could partially explain the cardioprotective role of OO.

## 2. The Platelets as Central Cellular Effectors of Chronic Diseases 

Platelets are the smallest blood cells (average diameter 2–5 μm) and they circulate in numbers ranging from 150 to 350 × ${\mathrm{10}}^{9}$/L in healthy subjects. They are anucleate, discoid, fragments of megakaryocytes which in turn are derived from pluripotent stem cells in the bone marrow. Platelets are characterized by a rich granule system consisting of α-granules, dense granules and lysosomes. Upon activation platelets secrete α-granules and dense granules along with granules’ content comprising of a milieu of bioactive components (coagulants, anticoagulants, fibrinolytic proteins, chemokines, adhesion proteins, angiogenic factors, immune mediators, ions, biogenic amines, nucleotides). Platelets are also characterized by an extensive network of surface glycoproteins and other integral proteins by which platelets interact with each other but also with leukocytes, endothelium, subendothelial proteins and cancer cells [15]. Through the secretion of pleiotropic mediators and growth factors, the heterotypic interactions with other cells, their responsiveness to inflammatory stimuli through Toll-like receptors and the induction of neutrophil extracellular traps (NETs), platelets have a central role in haemostasis, thrombosis, inflammation, antimicrobial defense, neurological disorders, tumor growth and metastasis [9, 16].

The dysregulation of the platelet-endothelium crosstalk is the main mechanism underlying the involvement of activated platelets in vascular complications. A healthy, intact, endothelium prevents adhesion and aggregation of platelets by secreting physiological, endogenous antiplatelet agents such as prostacyclin and nitric oxide (NO). However, upon the physical disruption of endothelium, the exposure of subendothelial matrix proteins (von Willebrand Factor, vWF, collagen-COL) triggers the adhesion and activation of platelets to endothelium through their binding to membrane glycoproteins which is the first step of the physiological role of platelets in primary haemostasis [16]. However, a chronic, subclinical, activation of platelets due to metabolic, immune or redox reasons may contribute to atherosclerosis progression in several ways. Activated platelets can adhere to an intact endothelium releasing pro-atherogenic molecules and microparticles. They can promote oxidation of low density lipoprotein (LDL) particles while at the same time they are activated by oxidized LDL (oxLDL) in a positive feedback loop. Taking into account the widely accepted theory of endothelial inflammation as the leading cause of atherosclerosis, it should be mentioned that several antiplatelet drugs (aspirin, clopidogrel) have known anti-inflammatory properties. In addition, heterotypic interactions of platelets with leukocytes promote their recruitment to inflamed endothelium propagating atherogenesis [17].

An atherosclerotic endothelium is prone to arterial thrombotic disease leading to MI and ischemic stroke. The crucial role of platelets in atherothrombosis has been confirmed by epidemiological studies showing the ability of anti-platelet drugs, mainly aspirin, to prevent MI, cardiovascular death, ischemic stroke and total mortality as a secondary prevention [18]. While the role of antiplatelet therapy as a secondary prevention of ischemic diseases is well established, its role in the primary prevention is still debated. This is partly because of the conflicting results of the clinical studies [19, 20] but also because of the increased risk for bleeding.

Several pro-clinical and clinical studies have also shown that platelets play a central role in malignancy and metastatic potential of cancer cells. The crosstalk between platelets and malignant cells in a reciprocal way leads to the progression of malignant lesions but also to cancer-induced thrombosis. Chronic activation of platelets drives immune cells to the site of tumor sustaining the development of malignant lesions. Platelets contribute to an invasive phenotype of cancer cells and protects them during their presence in the circulation conferring by this way to their metastatic potential. On the other hand, cancer cells can induce platelet aggregation, activation and release of their granule content among which growth and angiogenic factors support tumor survival and promotion [9]. Randomized clinical trials with aspirin have shown that doses of 300 mg/day are effective in the primary prevention of colon-rectal cancer [21] and less efficient for other types of cancer [22, 23]. However, recent randomized controlled trials failed to show a protection of a low dose of aspirin for overall cancer incidence and cancer-related mortality [24]. Similar conflicting results were obtained when dual antiplatelet therapy with aspirin plus purinergic receptor, P2Y12 inhibitors was studied [25, 26].

Chronic activation of platelets is also a risk factor for neurological disorders such as Alzheimer’s disease (AD). Higher platelet activation is associated with faster cognitive decline in AD [27] and CAD patients [28]. Activated platelets can metabolize amyloid-beta (Aβ) precursor protein into its metabolites contributing by this way to the deposition of Aβ while the involvement of platelet to chronic vascular inflammation is another mechanism that may link activated platelets with AD [29].

Type 2 diabetes mellitus (T2DM) is probably the most characteristic non-communicable disease which is characterized by hyperactive and dysfunctional platelets while at the same time is a risk factor for all the aforementioned chronic diseases. The diabetic platelet is characterized by an increased platelet turnover, increased intracellular ${\mathrm{Ca}}^{2+}$, upregulation of P2Y12 signaling and oxidative stress, increased P-selectin and glycoprotein expression, impaired response to NO and prostacyclin, resistance to aspirin, decreased membrane fluidity. The main drivers of these cellular abnormalities are the metabolic disturbances characterizing T2DM such as insulin resistance, hyperglycemia, diabetic dyslipidemia, obesity and subclinical inflammation [30, 31].

It is therefore obvious that chronically activated, dysfunctional platelets mediate the progression of the majority of non-communicable diseases in a pleiotropic fashion. Therefore, antiplatelet therapy remains an attractive therapeutic means which however hasn’t reached the expected targets according to the preclinical studies. A significant proportion of the patients receiving antiplatelet therapy acquire some sort of resistance to it while at the same time bleeding remains an important side effect. It is therefore obvious that the consumption of foods demonstrating antiplatelet activity may be a less drastic but on the other hand a more sustainable way of achieving daily antiplatelet therapy, either alone or in combination with antiplatelet drugs.

## 3. Olive Oil—Constituents and Health Properties

Olive oil is the oil obtained from the fruit of olive trees (*Olea europaea L.*). According to the Annex VII (Part VIII) of Regulation (EC) No 1308/2013 of the European Union, OOs can be classified into 8 quality categories, namely extra virgin olive oil (EVOO), virgin olive oil (VOO), OO, olive pomace oil, lampante, refined olive oil, refined-olive pomace oil and crude pomace OO. Among them the first four types of OOs are the edible ones. EVOO has the highest quality in terms of organoleptic characteristics, low acidity (<0.8%) and it is obtained directly from the fruit by mechanical means only. VOO is similarly obtained by mechanical means only but it is inferior to EVOO mainly to its higher acidity (<2%) and lower physicochemical and sensory properties. OO is a blend of refined olive oil with small amounts of either EVOO or VOO. It is inferior to EVOO and VOO and its acidity should not exceed 1%. Finally, olive-pomace oil is a mixture of refined olive pomace oil with small amounts of EVOO or VOO. It has the lowest quality of all edible oils, its acidity should not exceed 1% [32].

All OOs are composed of a saponifiable and an unsaponifiable fraction. The saponifiable fraction accounts for almost 98% of OO composition and it consists of triglycerides, diglycerides, monoglycerides and free fatty acids (FA). It is characterized by a high percentage of MUFA, mainly oleic acid (55–83%), and lower amounts of polyunsaturated fatty acids (PUFA), mainly linoleic acid (3–21%) and saturated fatty acids (SFA) (palmitic acid: 8–20% and stearic acid: 0.5–5%). However, the unsaponifiable fraction of OOs, although low in quantity, confers the beneficial biological properties of OO due to the presence of a milieu of over 250 biologically active phytochemicals. These include squalene (800–8000 mg/Kg), β-carotene and lutein (4–10 mg/Kg), sterols (1000–3000 mg/Kg), triterpenic compounds (200–300 mg/Kg), tocopherols/tocotrienols (250–350 mg/Kg) and polyphenols (200–1500 mg/Kg) [33].

Olive oil phenolics are secondary plant metabolites, produced by olive cells to combat pests and bacteria. They are responsible for the aroma and flavor of OOs but they also confer oxidative stability to the oil. Tocopherols/tocotrienols are the main lipid soluble phenolic compounds while at least 40 hydrophilic phenolic compounds have been identified in OO. According to their chemical structure can be classified into phenolic acids (photocatechouic, vanillic acid), phenolic alcohols (hydroxytyrosol-HT, tyrosol-TYR), secoiridoids (oleuropein OE, oleuropein aglycone OEA, ligstroside aglycone), flavonoids (luteolin-LU, apigenin-AP), lignans and hydroxy-isochromanes. EVOO contains the highest amount of total phenolics (200–800 mg/Kg), followed by VOO (60–350 mg/Kg) and refined OO (60–200 mg/Kg). In terms of biological properties and quantity, TYR, HT, their secoiridoid derivatives oleacein and oleocanthal, OEA and ligstroside are the most important phenolic compounds in OO [32, 33, 34].

Olive oil is probably the main cardioprotective component of the MD according to the results of observational and dietary intervention studies [35]. The emblematic PREDIMED study showed that the adoption of a MD type of diet with EVOO as the main fat source resulted in a 30% reduction of CVDs (acute MI, stroke, death from CVD causes) [36]. The cardioprotective properties of OO were initially attributed to its high MUFA content. However, recent meta-analyses of cohort studies have shown that the dietary intake of either total fat, SFA, MUFAs or PUFA were not associated with the risk of cardiovascular disease while the dietary source (animal or vegetable) of MUFA may play a role on the association of MUFA and CVDs [37, 38]. Therefore, nutritionists seeked alternative explanations for the beneficial properties of EVOO. Among all phytochemicals of OO, its unique phenolics seems to be responsible for the majority of its cardioprotective properties, although the research is ongoing. Numerous *in vitro* and preclinical studies have demonstrated the pleiotropic actions of phenol extracts or individual EVOO phenolics on inflammation, endothelial dysfunction, coagulation, dyslipidemia and oxidative stress [32, 39]. These pathological mechanisms underlie the majority of chronic disease and their regulation by OO phenolics can partly explain the health promoting properties of OO. Although certain molecular targets are linked to certain phenolics it seems that they act in a more general way as nutritional hormetins triggering lifelong adaptive processes preventing by this way the biological aging [40].

Taking into account the central role of platelets in the pathogenesis of chronic diseases, especially CVDs, and the cardioprotective properties of OO as part of the MD, in the next chapters of this review we investigate the relationship of OO phenolics with platelet functions as another mechanistic explanation of the OOs’ health benefits.

## 4. Search Methodology

We searched the literature in PubMed and Scopus databases until March 2022 using a combination of the following keywords: olive oil, virgin olive oil, extra virgin olive oil, phenolics, polyphenols, phenols, tyrosol, hydroxytyrosol, oleocanthal, oleuropein, oleacein, secoiridoids, platelets, platelet aggregation, platelet adhesion, platelet rich plasma (PRP). We included *in vitro*/*ex vivo* studies, animal studies and dietary intervention studies in humans. At least one assay of platelet functionality should be included in these studies. In cell and animal studies, the active ingredients should be either EVOO or VOO with a known amount of total phenolics, isolated phenolic extracts with some sort of phenolic characterization, isolated or synthetic phenolic molecules and synthetic derivatives of them. For the human studies, we selected dietary intervention trials where at least one intervention group included the administration of EVOO, VOO with a known amount of total phenolics. We have to mention that in many articles OO was administered as a placebo to clinical trials investigating the health properties of PUFAs. Those studies were excluded from this review since the OOs were not characterized for their phenolic composition.

## 5. Effect of Phenolic Compounds/Extracts of Olive Oil on Platelet Functions—*In Vitro* Studies

Cellular studies in isolated platelets is a convenient way for screening the antiplatelet properties of bioactive compounds and investigating the possible mechanisms by which these compounds exert their antiplatelet effects. The characteristics and the main outcomes of the *in vitro* studies exploring the modulatory role of OO phenolic extracts on platelet functions are presented in Table 1 (Ref. [41, 42, 43, 44, 45, 46, 47, 48, 49, 50, 51, 52, 53]).

**Table 1.** **Effect of phenolic compounds/extracts of OO on platelet functions *in vitro***.

Reference	Platelet source	Platelet preparation	Tested compounds/extracts	Assays of platelet function	Results
Petroni A, 1995 [46]	Healthy volunteers	PRP, WB	HT, OE, LU, AP, phenol-rich extract from WW	COL-induced aggregation	HT inhibited COL-induced aggregation (${\mathrm{IC}}_{50}$: 67 μΜ)
				ADP-induced aggregation	HT inhibited ADP-induced aggregation (23 μΜ)
				COL-induced ${\mathrm{TxB}}_{2}$ production in PRP	HT (400 μΜ) inhibited COL-induced (81%) and THR-induced (84%) ${\mathrm{TxB}}_{2}$ production in PRP
				THR-induced ${\mathrm{TxB}}_{2}$ production in PRP	HT (400 μΜ) inhibited ${\mathrm{TxB}}_{2}$ (94%) and 12-HETE accumulation in serum during clotting
				${\mathrm{TxB}}_{2}$ and 12-HETE during clotting	Order of inhibitory potential of COL-induced aggregation: aspirin (100 μΜ, 100%), WW extract (20 ppm GA equiv. around 100 μΜ, 80%), ΗΤ (100 μΜ, 55%), LU (100 μΜ, 23%), ΟΕ (100 μΜ, 11%), AP (100 μΜ, 0%)
Togna GI, 2003 [41]	Healthy volunteers	PRP	1-(3- methoxy-4-hydroxy-phenyl)-6,7-dihydroxy-isochroman	COL, ADP, SA-induced aggregation	Both compounds inhibited COL-and SA-induced aggregation and ${\mathrm{TxB}}_{2}$ release (0.1–20 μΜ)
			1-phenyl-6,7-dihydroxy-isochroman	COL, ADP, SA-induced ${\mathrm{TxB}}_{2}$ production in PRP	Both compounds inhibited COL, THR-induced AA release (50–200 μΜ, 5–25%)
				COL, THR-induced AA release	
Turner R, 2005 [42]	Healthy volunteers	WB	TYR, HT, OE, homovanillic alcohol	COL-induced aggregation	No effect of all phenolics (100 μΜ) on COL-induced aggregation
Fragopoulou E, 2007 [44]	Rabbits	Washed platelets	TYR and acetylated derivatives	PAF-induced aggregation	TYR inhibited PAF-induced aggregation (${\mathrm{IC}}_{50}$: 2 mM). Its acetylated derivatives are more potent inhibitors (0.01–0.04 mM)
Dell’Agli M, 2008 [49]	Healthy volunteers	Washed platelets	Phenolic extracts from olive oils with a high and low phenol content. The phenolic extracts contained HT, TYR, OEA,QU, LU and AP	THR-induced aggregation	Inhibition of THR-induced aggregation (${\mathrm{IC}}_{50}$s: 1–7 μg/mL for High Phenol Extracts and 11 μg/mL for Low Phenol Extracts)
		Platelet lysates	OEA, LU, AP, TYR, HT, OE, HVA	cAMP PDE activity	Inhibition of THR-induced aggregation at 10 μΜ (OEA 75%, LU 23%, HT 11%, TYR 10%, HVA 7%, AP 0%)
				cGMP PDE5A1 activity	Inhibition of cAMP PDE activity (${\mathrm{IC}}_{50}$s: 14–33 μg/mL for High Phenol Extracts, 28 μg/mL for Low Phenol Extracts, 89 μΜ OEA, 1 μΜ LU, 4 μΜ AP, HT, TYR and HVA were inactive up to 100 μM)
					Lack of inhibition of PDE5A1 by phenol extracts
					Inhibition of PDE5A1 activity at 50 μΜ ( LU 51%, HT 41%, AP 21%, OEA 9%, TYR 0%)
Correa J, 2009 [50]	Healthy volunteers	WB, PRP, PRP plus erythrocytes, PRP plus leukocytes	HT and HTA	COL, ADP, AA-induced aggregation in WB and PRP	Inhibition of COL-induced aggregation in WB (${\mathrm{IC}}_{50}$s: HT 195 μM, HT acetate 26 μM)
				Platelet production of ${\mathrm{TxB}}_{2}$ in WB	Inhibition of ADP-induced aggregation in WB (${\mathrm{IC}}_{50}$s: HT 738 μM, HT acetate 94 μM)
					Inhibition of AA-induced aggregation (${\mathrm{IC}}_{50}$s: HT 197 μM, HT acetate 40 μM)
					Inhibition of ${\mathrm{TxB}}_{2}$ production (${\mathrm{IC}}_{50}$s: HT 63 μM, HT acetate 5) μM
Zbidi H, 2009 [52]	Healthy volunteers, Diabetic patients	Washed platelets	HT, OE	THR-induced aggregation	OE (10–300 μM) slightly inhibited THR-induced aggregation (20%)
				THR-induced ${\mathrm{Ca}}^{2+}$ mobilization	OE (10 μM) inhibited thrombin-induced ${\mathrm{Ca}}^{2+}$ release from the intracellular stores (44%) and ${\mathrm{Ca}}^{2+}$ entry (32%). The effect was greater in diabetic platelets. HT less efficient than OE
				THR-induced protein tyrosine phosphorylation	OE (10 μM) and HT (100 μM) inhibited THR-induced protein tyrosine phosphorylation both in healthy donors and diabetic subjects
De Roos, 2011 [47]	Healthy volunteers	PRP	Alperujo extract (containing HT and DHPG), HT, DHPG	COL, TRAP-induced aggregation in PRP	Inhibition of COL- and TRAP-induced aggregation by alperujo extract (5–28%, 5–16%)
				ADP, TRAP-induced expression of P-selectin and fibrinogen on the surface of PLTs (Flow cytometry)	Synergistic antiaggregating effect of HT (40 mg/L) and DHPG (5 mg/L) (COL: 12%, TRAP: 16%)
				ADP-induced changes in proteome	Inhibition of ADP, TRAP-induced expression of P-selectin and fibrinogen by alperujo extract (9–12%)
					Differential regulation of nine proteins by the alperujo extract upon ADP-induced platelet aggregation. The proteins are involved in regulation of platelet structure and aggregation, coagulation and apoptosis, and signalling by integrin αIIb/β3
Reyes JJ, 2013 [51]	Healthy volunteers	WB	HT and HT alkyl ether derivatives	COL, AA, ADP-induced aggregation	HT inhibited COL (${\mathrm{IC}}_{50}$: 193 μM), AA (${\mathrm{IC}}_{50}$: 190 μM), ADP (${\mathrm{IC}}_{50}$: 716 μM)-induced aggregation. HT alkyl ether derivatives are more potent inhibitors of plt aggregation
Rubio-Senent F, 2015 [53]	Healthy volunteers	PRP	PE obtained from steam treatment of alperujo	COL,TRAP-induced aggregation	HTA (510 μM):
			Polymeric phenolic fractions (PPF)		inhibition of 3 and 5 μg/L COL (38,27%). Inhibition of 25 μM TRAP—induced platelet aggregation by 37 %
			HT, DHPG, HTA		PPF (100 mg/L): inhibition of 3 and 5 μg/L COL (23,13%). Inhibition of 25 μM TRAP—induced platelet aggregation by 22 %
					PE (500 mg/L): inhibition of 3 and 5 μg/L COL (52,40%). Inhibition of 25 μM TRAP—induced platelet aggregation by 19%
					Mixtures of HT + HTA are more potent than the compounds tested alone
Carnevale R, 2018 [45]	Healthy volunteers	PRP	Italian EVOOs (149–392 mg/L GAE)	NOX2 activity	The AA-induced upregulation of NOX2 and $H_{2}$$O_{2}$ production was significantly inhibited by EVOOs
				Platelet $H_{2}$$O_{2}$	The content of both vitamin E and total polyphenol inversely correlated with NOX2 activation and directly with $H_{2}$$O_{2}$ scavenging property
Mizutani D, 2020 [48]	Healthy volunteers	PRP	HT, OE	COL-induced aggregation and size of plt aggregates	HT and OE (30–100 μM) inhibited COL-induced aggregation
				COL-induced secretion of PDGF-AB	HT (100 μM) and OE (400 μM) significantly increased the formation of small aggregates but decreased the formation of large aggregates
				COL-induced secretion of sCD40L	HT (100 μM) and OE (500 μM) reduced the COL-induced secretion of PDGF-AB (60%, 80%) and the release of sCD40L (55%, 90%)
				COL-induced phosphorylation of HSP27	HT (100–150 μM) and OE (500 μM) attenuated the COL-induced phosphorylation of HSP27
				COL-induced release of phosphorylated HSP27	HT (100 μM) and OE (500 μM) suppressed the COL-induced release of phosphorylated HSP27 (75%, 90%)
Pathania A, 2021 [43]	Healthy volunteers	PRP lysate	HT	MAO-B activity	HT inhibited MAO-B activity (${\mathrm{IC}}_{50}$: 7.78 μM)

AA, Arachidonic Acid; ADP, Adenosine diphosphate; AP, Apigenin; cAMP, cyclic 3’ 5’ adenosine monophosphate; cGMP, Cyclic guanosine monophosphate; COL, Collagen; 12-HETE, 12-hydroxy-5,8,10,14-eicosatetraenoic acid; DHPG, 3,4-dihydroxyphenylglycol; EVOO, Extra virgin olive oil; GAE, Gallic acid equivalent; HSP, Heat Shock Protein; HT, Hydroxytyrosol; HTA, Hydroxytyrosol acetate; HVA, Homovanillic alcohol; LU, Luteolin; MAO, Monoamine oxidase; NOX2, NADPH oxidase 2; OE, Oleuropein; OEA, Oleuropein aglycon; PAF, Platelet-activating factor; PDE, phosphodiesterase; PE, Phenolic extracts; PLT, platelet; PPF, Polymeric phenolic fractions; PRP, Platelet rich plasma; QU, quercetin; THR, Thrombin; TRAP, Thrombin Receptor Activating Peptide; ${\mathrm{TxB}}_{2}$, Thromboxane B2; TYR, Tyrosol; SA, Sodium Arachidonate; WB, Whole Blood; WW, waste water.

The majority of the studies have used PRP or whole blood (WB) from healthy human donors as the source of platelets. The most common platelet assays that are utilized in those studies are agonist-induced platelet aggregation and thromboxane B2 (${\mathrm{TxB}}_{2}$) production. The endogenous agonists more frequently used for the *ex vivo* activation of platelets were COL, adenosine diphosphate (ADP), thrombin (THR) and arachidonic acid (AA).

The studies so far have shown that several phenolic compounds and extracts of OO are able to inhibit platelet activation. The strongest evidence exists for HT since many cell studies have demonstrated its anti-aggregating potential. HT was able to inhibit COL-, ADP- and Thrombin Receptor Activating Peptide (TRAP)-induced platelet aggregation in human PRP [46, 47, 48], THR-induced aggregation in washed human platelets [49], COL-, ADP- and AA-induced aggregation in WB of healthy donors [50, 51]. Only one study could not demonstrate the inhibitory potential of HT at 100 μM against COL-induced platelet aggregation in the WB of healthy donors. Among the other phenolic compounds, OE [46, 48, 49, 52], OEA [49], TYR [49], deacetoxy oleuropein aglycone (DHPG) [47, 53] and hydroxytyrosol acetate (HTA) [50, 53] have shown anti-aggregating properties in at least one study. The majority of studies pre-incubated platelets for 10 min before the addition of the aggregating agents since this time seems to maximize inhibition of platelet aggregation [46].

Phenol-rich extracts from OOs [49], waste water (WW) [46], alperujo (a solid by-product of OO extraction) [47] and steam-treated alperujo [53] also demonstrate potent antiplatelet properties. Phenolic extracts seems to have a better antiplatelet action compared to pure compounds probably due to the synergistic effect of the phenolics. A phenolic extract from WW containing a mixture of phenolic compounds with a concentration comparable to pure HT (100 μM) has a better anti-aggregating activity compared to isolated compounds [46]. In addition, an alperujo extract containing HT and DHPG has a small but significant antiaggregating activity against COL and TRAP-induced aggregation of human platelets while the isolated compounds have negligible effect [47]. Future studies should investigate whether OO by-products could serve as a rich, cheap, source of polyphenols able to formulate nutraceuticals and supplements with cardioprotective properties [54].

A couple of studies have shown that the ability of the phenolic compounds to inhibit platelet aggregation depends on the aggregating agent. Two isochromans found in EVOO inhibited the platelet response to AA and COL, but not to ADP. The authors of this study support the hypothesis that these isochromanans, through their radical scavenging activity can inhibit only the initial, redox sensitive phases of platelet activation, induced by COL and AA [41]. HT, HTA and aspirin showed a lower inhibition (higher ${\mathrm{IC}}_{50}$ values) of ADP-induced platelet aggregation compared to COL-induced platelet inhibition probably because these phenolics interact with the COL-induced eicosanoid production rather than the lowering of ${\mathrm{Ca}}^{2+}$ which mainly mediates ADP-induced platelet aggregation [50].

The ability of the phenolic compounds to inhibit platelet activation depends on the biological source of platelets indicating that phenolics can interact with platelet either directly (PRP) or indirectly through the production of platelet modulating agonists/antagonists from other blood cells. For example, HT and OE could not inhibit COL-induced platelet aggregation in WB [42] while the same molecules inhibited COL-induced aggregation in human PRP [46]. This was demonstrated more emphatically in the study of Correa *et al*. [50], which showed that HTA is a better inhibitor of HT in WB while they showed similar inhibitory properties in PRP. Both phenolics could inhibit platelet thromboxane $A_{2}$ (${\mathrm{TXA}}_{2}$) production but HTA could also indirectly inhibit platelet aggregation in WB by stimulating NO production by leukocytes [50].

The structure of the phenolic compounds, seems to determine their antiplatelet properties. In a study comparing different phenolic compounds HT was a better inhibitors of COL-induced platelet aggregation compared to OE, LU, AP [46]. Similarly, in the study of Togna *et al*. [41], only the most lipophilic isochroman derivatives showed a significant inhibitory potential against COL-induced aggregation. Whether the clinical condition of the donor could affect the antiplatelet properties of the phenolic compounds has not been investigated. Just one study showed that OE has a slightly better antiaggregating activity in normal platelets compared to diabetic ones at low concentrations (10 μM) [52].

The ability of OO phenolics to inhibit platelet aggregation can be attributed to several mechanisms but the inhibition of ${\mathrm{TxA}}_{2}$ production from eicosanoids seems to be the most relevant one. HT and HTA can inhibit COL- and THR-induced ${\mathrm{TxB}}_{2}$ production in PRP and WB of healthy volunteers and the production of ${\mathrm{TxB}}_{2}$ and 12-hydroxy-5,8,10,14-eicosatetraenoic acid (12-HETE) during clotting [46, 50]. Taking into account that HT and HTA can also inhibit prostacyclin production in WB it seems than the inhibition of ${\mathrm{TxA}}_{2}$ production is due to inhibition of cyclooxygenase rather than TxB synthetase [50]. OO isochromans can also inhibit arachidonic acid mobilization from platelet membrane phospholipids induced by THR and COL indicating a direct inhibition of PLA2 [41]. OE and HT can also interfere with THR-induced calcium mobilization. This effect was greater in platelets from T2DM subjects. The antioxidant properties of OE and HT could explain the reactive oxygen species (ROS)-mediated entry of ${\mathrm{Ca}}^{2+}$ into activated platelets [52]. OE (10 μM) and HT (100 μM) could also inhibit THR-induced protein tyrosine phosphorylation both in healthy donors and diabetic subjects [52]. Phenolic extracts from OO with a high and low phenolic content were able to inhibit the degrading enzyme of cyclic 3’ 5’ adenosine monophosphate (cAMP), cAMP phosphodiesterase, preventing by this way the lowering of intraplatelet cAMP levels, which is a crucial mechanism of platelet activation [49]. Whether the antioxidant properties of phenolics could explain their antiplatelet actions is again a controversial and not well-studied issue. The radical scavenging activities of a series of OO phenolic compounds are not correlated with their ability to inhibit platelet aggregation in PRP of healthy volunteers [46]. On the other hand, Italian EVOOs could downregulate the activation of platelet nicotinamide adenine dinucleotide phosphate (NADPH) oxidase (NOX) activation induced by AA. NOX can induce the production of ROS which in turn contribute to the production of the platelet agonist 8-iso-PGF2 alpha. The polyphenol and tocopherol content of the EVOOs correlated with the inhibitory activity probably through the scavenging of $H_{2}$$O_{2}$ [53]. HT (100 μM) and OE (500 μM) could also reduce the secretion of pro-atherogenic molecules from platelet granules such as platelet-derived growth factor (PDGF)-AB and soluble CD40L (sCD40L) [48] and suppress the COL-stimulated phosphorylation of heat shock protein 27 (HSP27) in human platelets, leading to the reduction of phosphorylated-HSP27 release which is able to activate the release of pro-inflammatory cytokines from macrophages [48]. HT is also inhibitor of the platelets’ monoamine oxidase isoform (MAO)-B. Taking into account that the primary structure of MAO-B in platelets and brain is almost identical this indicates a possible role of HT in alleviating Parkinson disease symptoms [43]. Finally, proteomic analysis revealed that phenolic-rich extracts, specifically an alperujo extract containing HT and DHPG, have acute effects on platelet proteome regulating multiple pathways involved in platelet aggregation, coagulation, signalling and apoptosis [47].

Several studies have shown that the derivatization of OO phenolics may lead to molecules with better antiplatelet activity. The acetylated derivatives of TYR, which are also found in olives and OOs are two orders of magnitude more potent inhibitors of platelet-activating factor (PAF)-induced aggregation in washed rabbit platelets than tyrosol itself [44]. Acetylated HT has a better inhibitory activity against COL, ADP, AA-induced aggregation in WB and PRP compared to HT [50]. Hydrophobic derivatives of HT (alkyl HT ethers) are better inhibitors of COL, AA, ADP-induced platelet aggregation in WB of healthy volunteers although they similarly inhibited ${\mathrm{TxB}}_{2}$ production [51].

In conclusion, OO phenolic compounds, mainly HT and OE show antiplatelet properties affecting multiple pathways of platelet activation. However, the concentrations used in the *in vitro* experiments can not be achieved in blood even under postprandial conditions. Therefore, *in vitro* experiments may facilitate a rapid screening and may give some hints on the mechanism involved in the antiplatelet properties of OO phenolics but from a nutritional point of view the outcomes of these experiments should be translated to real clinical settings with caution.

## 6. Effect of Phenolic Compounds/Extracts of OO on Platelet Functions—Animal Studies

The studies utilizing animal models to investigate the antiplatelet properties of EVOO, VOO, phenolic extracts or pure phenolic compounds are shown in Table 2 (Ref. [55, 56, 57, 58, 59, 60, 61]). The majority of the studies used rats as the preferred animal species while only one study used New Zealand rabbits. The pathological phenotypes induced by the researchers were either atherosclerosis (induced by an atherogenic diet) [55], diabetes (induced by streptozotocin) [56, 57] while the rest of the intervention studies were conducted in healthy animals. The investigators administered either VOO [55, 56, 58], EVOOs with different concentrations of phenolics [59], HT [57, 60], HTA [60], DHPG [57] and alkyl ether derivatives of HT [61]. The interventions lasted from 0 days (acute administration) to 3 months while the experimental oils/molecules were administered by oral gavage in the majority of the studies.

**Table 2.** **Dietary intervention with OO phenolics/extracts in animal models**.

Reference	Animal Model	Intervention groups	Administration	Duration of intervention	Assays of platelet function/composition	Results
De La Cruz, 2000 [55]	White New Zealand rabbits	NLD: Normolipemic standard diet (34% SFA)	Solid food mixed with VOO	6 weeks	ADP- and COL-induced platelet aggregation in WB	Increased EC50 values for ADP-(3.3 fold) and COL-(2.5 fold) aggregation in the SFAED + OLIV group compared to SFAED group
		SFAED: Atherogenic diet containing 48% SFA and 1% cholesterol			${\mathrm{TxB}}_{2}$ production during clotting	67% lower ${\mathrm{TxB}}_{2}$ production in SFAED + OLIV compared to SFAED
		NLD + OO: Normolipemic standard diet + 15% VOO			AA-induced lipid peroxides production in PRP	Lower lipid peroxides (57%) in SFAED + OLIV compared to SFAED
		SFAED + OO: Atherogenic diet + 15% VOO			Platelet-Subendothelium interactions	Lower thrombus formation in the subendothelium in the SFAED + OLIV group compared to SFAED group
Priora R, 2008 [59]	Rats (adult male Sprague-Dawley)	ROO: (1.25 mL/Kg BW) containing 8.4 mg/L MPC	Oral gavage	Acute or 12 days	ADP-induced platelet aggregation in PRP	Acute inhibition of platelet sensitivity to ADP only in the HC group
		LC: EVOO with low minor polar compounds (1.25 mL/Kg BW) containing 213 mg/L MPC				Both LC and HC inhibited ADP-induced platelet aggregation after 12 d administration
		HC: EVOO enriched with minor polar compounds (1.25 mL/Kg BW) containing 510 mg/L MPC				Only platelet reversible aggregation was inhibited by HC
González-Correa J, 2007 [58]	Rats (male Wistar)	Control: Saline	Oral gavage	30 days	COL-induced aggregation in WB	VOO 0.25 and 0.5 mL/Kg/day inhibited COL-induced aggregation by 36 and 47%
		VOO 0.25 mL/Kg/day			COL-induced ${\mathrm{TxB}}_{2}$ production in WB	VOO 0.25 and 0.5 mL/Kg/day inhibited COL-induced ${\mathrm{TxB}}_{2}$ production by 19 and 26%
		VOO 0.5 mL/Kg/day				
		VOO contains a relatively low amount of phenols (0.19 mmol/Kg ortho-diphenols)				
González-Correa J, 2008 [60]	Rats (male Wistar)	16 groups of animals (6 animals per group)	Oral gavage	7 days	COL-induced aggregation in WB	Inhibition of COL-induced aggregation by HT (${\mathrm{ID}}_{50}$: 48 mg/Kg/day), HTA (${\mathrm{ID}}_{50}$: 16 mg/Kg/day), ASA (${\mathrm{ID}}_{50}$: 2.4 mg/Kg/day) in a dose-dependent manner
		Control group (isotonic saline solution)			${\mathrm{Ca}}^{2+}$-induced ${\mathrm{TxB}}_{2}$ production in WB	Weak inhibition of ${\mathrm{Ca}}^{2+}$-induced ${\mathrm{TxB}}_{2}$ production by HT (30%) and HTA (37%) at a dose of 100 mg/Kg/day
		Six groups treated with HT (1, 5, 10, 20, 50, and 100 mg/Kg/day)				
		Six groups treated with HT-				
		AC (1, 5, 10, 20, 50, and 100 mg/Kg/day)				
		Three groups treated with ASA (1, 5, and 10 mg/Kg/day).				
De La Cruz, 2010 [56]	Rats (male Wistar)	Control, non-diabetic group	Oral gavage	3 months	COL-induced aggregation in WB	ASA reduced maximum intensity of platelet aggregation by 51%, VOO by 41 % and ASA plus
		Streptozotocin-induced diabetes			COL-induced ${\mathrm{TxB}}_{2}$ production in WB	VOO by 81 % compared to untreated diabetic rats
		Streptozotocin-induced diabetes + ASA (2 mg/Kg/day)				ASA reduced *ex vivo* ${\mathrm{TxB}}_{2}$ production by 98%, VOO by 46 % and ASA plus
		Streptozotocin-induced diabetes + VOO (0.5 mL/Kg/day)				VOO by 98 % compared to untreated diabetic rats
		Streptozotocin-induced diabetes + ASA (2 mg/Kg/day) + VOO (0.5 mL/Kg/day)				
		VOO contained 250 mg/Kg total phenols				
Muñoz-Marín J, 2013 [61]	Rats (male Wistar)	Control group: Saline	Oral gavage	7 days	COL-induced aggregation in WB	The alkyl ether derivatives reduced maximum intensity of aggregation in a dose and structure dependent manner. The highest inhibition was achieved by hexyl derivative (59%)
		HT groups: 20 and 50 mg/Kg/day			${\mathrm{Ca}}^{2+}$-induced ${\mathrm{TxB}}_{2}$ production in WB	The alkyl ether derivatives reduced ${\mathrm{TxB}}_{2}$ production in a dose and structure dependent manner. The highest inhibition was achieved by hexyl ether derivatives at 20 and 50 mg/Kg/day (58 and 61%, respectively)
		HT ethyl ether: 20 and 50 mg/Kg/day				
		HT butyl ether: 20 and 50 mg/Kg/day				
		HT hexyl ether: 20 and 50 mg/Kg/day				
		HT octyl ether: 20 and 50 mg/Kg/day				
		HT dodecyl ether: 20 and 50 mg/Kg/day				
De La Cruz Cortés JP, 2021 [57]	Rats (male Wistar)	NDR: saline-treated non-diabetic rats	Oral gavage	2 months	COL-induced aggregation in WB	HT completely reversed the increase of maximum intensity of platelet aggregation observed in DR to levels lower than those observed in NDR
		Glycemic control rats			Urine 11-dH-${\mathrm{TxB}}_{2}$ levels	DHPG completely reversed the increase of maximum intensity of platelet aggregation observed in DR to levels lower than those observed in NDR in a dose dependent manner
		DR: Streptozotocin-induced, saline-treated, diabetic rats				Synergistic effect of HT and DHPG in inhibiting *ex vivo* platelet aggregation
		DR + HT: Diabetic rats treated with 5 mg/Kg/day HT				HT completely reversed the increase of urine 11-dH-${\mathrm{TxB}}_{2}$ levels observed in DR
		DR + DHPG-0.5: Diabetic rats treated with 0.5 mg/Kg/day DHPG				DHPG partially reversed the increase of urine 11-d-H-${\mathrm{TxB}}_{2}$ levels observed in DR
		DR + DHPG-1: Diabetic rats treated with 1 mg/Kg/day DHPG				
		DR + HT + DHPG-0.5: Diabetic rats treated with 5 mg/Kg/day HT plus 0.5 mg/Kg/day DHPG				
		DR + HT + DHPG-1: Diabetic rats treated with 5 mg/Kg/day HT plus 1 mg/Kg/day DHPG				

AA, Arachidonic Acid; ADP, Adenosine diphosphate; ASA, Acetylsalicylic acid; COL, Collagen; DHPG, 3,4-dihydroxyphenylglycol; DR, diabetic rats; HC, high MPC concentration; HT, Hydroxytyrosol; HTA, hydroxytyrosol acetate; ${\mathrm{ID}}_{50}$, Inhibitory dose for 50% inhibiton; LC, low MPC concentration; MPC, minor polar compounds; NLD, normolipemic diet; NDR, Nondiabetic rats; PLT, platelet; SFAED, saturated fatty acid-enriched diet; ROO, Refined Olive Oil; ${\mathrm{TxB}}_{2}$, Thromboxane; VOO, virgin olive oil; WB, Whole Blood.

The most important outcome of these studies is that the platelet inhibition by OO phenolics is accompanied by improvements of the pathological phenotypes. OO could slow atherosclerosis progression concomitantly with inhibition of platelet hyperreactivity. Specifically, when an atherogenic diet was accompanied with the supplementation of VOO (15%) to New Zealand rabbits the atherosclerotic lesions of the arteries were improved concomitantly with the lowering of platelet aggregation, ${\mathrm{TxB}}_{2}$ and lipid peroxide production [55]. VOO could also attenuate *ex vivo* brain damage concomitantly with inhibition of platelet hyperreactivity. *Ex vivo* brain damage in fresh brain slices of rats after hypoxia-reoxygenation was attenuated with the administration of two doses of VOO and this improvement was accompanied with the lowering of *ex vivo* platelet aggregation and ${\mathrm{TxB}}_{2}$ production in rats. It seems that platelet inhibition could play a key role in the improvement of throbogenesis and brain ischemia and may play a role in the prevention of ischemic stroke [58]. Finally, VOO can act synergistically with acetylsalicylic acid (ASA) to inhibit vascular complications in the retina of diabetic rats. Administration of VOO (0.5 mL/Kg/day per os) attenuated the activation of platelet activity induced by diabetes (platelet aggregation, ${\mathrm{TxB}}_{2}$ production, reduction of aortic prostacyclin and NO production). This effect was accompanied by improvements of retinal ischemia measured as the retinal surface area occupied by peroxidase-permeable vessels [56].

The dosage of the phenolic compounds is a major determinant of their antiplatelet properties. HT and HTA inhibited COL-induced platelet aggregation in WB of rats in a dosage range of 1–100 mg/Kg/day. Their ability to inhibit platelet aggregation was positively correlated with the dosage of the phenolic compounds [60]. Priora *et al*. [59] compared the antiplatelet effects of 3 OOs with a similar fatty acid (FA) content but different amounts of minor polar compounds (MPC). They conducted both acute (one dose) and subacute (12 d) experiments demonstrating the superiority of the EVOO-enriched with MPC to inhibit platelet aggregation. The MPC contained HT, TYR, elenolic acid, OEA but mainly deacetoxy oleuropein aglycone (DACOLAG) and secoiridoid derivatives [59]. The synergism between phenolic compounds could explain the superiority of whole OOs or phenolic extracts against pure phenolic compounds to inhibit platelet activation. A recent study of De La Cruz Cortés *et al*. [57], clearly showed the synergistic effect of HT and DHPG in similar proportions found in EVOO to attenuate COL-induced platelet aggregation of diabetic rats and to spare prostacyclin synthesis by the aorta.

The investigation of the mechanisms underlying the antiplatelet effects of OO phenolics demonstrated once more the ability of VOO and its phenolics to inhibit ${\mathrm{TxB}}_{2}$ synthesis. The administration of VOO inhibited the *ex vivo* ${\mathrm{TxB}}_{2}$ production in an experimental model of atherosclerosis in rabbits [55], in healthy rats in a dose dependent manner [58] and in streptozotocin-induced diabetic rats [56]. HT and HTA, at a dosage scheme of 100 mg/Kg/day inhibited ${\mathrm{Ca}}^{2+}$-induced ${\mathrm{TxB}}_{2}$ synthesis in WB [60]. The ability of EVOO enriched with MPCs to inhibit only reversible platelet aggregation could be explained by the radical scavenging properties of phenolics since ROS mainly affect the activation of fibrinogen receptors in platelets but not the release of granules by platelets [59]. Another indirect mechanism of platelet inhibition is the stimulation of NO and prostacyclin synthesis by the endothelium. VOO stimulated prostacyclin synthesis in the aorta of rabbits fed an atherogenic diet [55]. VOO containing 250 mg/Kg total phenols partially reversed inhibition of ${\mathrm{Ca}}^{2+}$-induced prostacyclin production by the aortas of streptozotocin-induced diabetic rats. This effect could not be observed by aspirin which inhibits aortic prostacyclin production due to endothelial cyclooxygenase (COX) inhibition [56]. HT and HTA were able to increase *ex vivo* NO synthesis in a manner proportionally similar to aspirin [60]. An alternative explanation for the indirect antiplatelet properties of phenolics is offered by Priora *et al*. [59] who showed that administration of EVOO rich in MPCs to rats is able to inhibit *ex vivo* ADP-induced platelet aggregation in PRP which is accompanied by a reduction of reduced homocysteine (rHcy) levels. Previous studies have shown that rHcy has pro-aggregating properties increasing ${\mathrm{TxB}}_{2}$ production in human platelets [62].

The study of De La Cruz *et al*. [56] has also demonstrated the synergistic effect of VOO containing 250 mg/Kg total phenols with aspirin in inhibiting platelet aggregation in streptozotocin-induced diabetic rats. Treatment of diabetic rats with both aspirin and VOO produces greater inhibition of ADP-induced platelet aggregation compared to the administration of aspirin and VOO separately. Both VOO and aspirin could inhibit TxB production by platelets due to COX inhibition while at the same time VOO phenolics act on aortic endothelium preventing the inhibition of vascular NO synthesis. This effect increases the prostacyclin to TxB ratio which is indicative of the thrombogenic potential of the vascular bed [56].

The animal studies have also proved the observation from cell studies that the increased lipophilicity of phenolic compounds and their derivatives is related with better antiplatelet properties. The administration of the acetylated derivative of HT to rats produces greater inhibition of COL-induced aggregation in WB (${\mathrm{ID}}_{50}$: 16 mg/Kg/day) compared to HT (${\mathrm{ID}}_{50}$: 48 mg/Kg/day) [60]. Muñoz-Marín J *et al*. [61] synthesized a series of alkyl ether derivatives of HT which are more lipophilic and stable. Compared to HT, the administration of its alkyl ether derivatives to rats reduced maximum intensity of COL-induced aggregation in a dose and structure dependent manner. The highest inhibition was achieved by hexyl derivative (59%). The alkyl ether derivatives were also better inhibitors of *ex vivo* ${\mathrm{Ca}}^{2+}$-induced ${\mathrm{TxB}}_{2}$ production in a dose and structure dependent manner. The highest inhibition was achieved by hexyl ether derivatives at 20 and 50 mg/Kg/day [61].

In conclusion, pre-clinical studies in animal models proved the ability of VOO and its phenolic compounds to inhibit *ex vivo* platelet activation and this inhibition was correlated with improvements of pathological phenotypes related to ischemic diseases. Although the dosage schemes for the VOO (0.25–1 mL/Kg/day) can be realistically achieved in the Mediterranean countries, this is not the case for the administration of pure phenolic compounds which are given in supraphysiological, pharmacological doses. Therefore, the outcomes of the latter studies have a value from a pharmacological point of view rather than from a nutritional perspective.

## 7. Dietary Interventions with EVOO and Phenolic Compounds/Extracts of OO in Humans 

Numerous studies have already used OO as a control/placebo to dietary interventions investigating the effect of other types of fats and oils (mainly PUFAs) on platelet activation. Olive oil was utilized as a biologically inert oil and as a source of MUFAs. In most cases, no characterization of the phenolic content was provided although, for the majority of the studies the usage of refined OO was implied. These studies are not included in this review since their design does not provide evidence for the ability of OO phenolics to inhibit platelet activation in humans.

A recent observational study demonstrated that obese patients who were frequent consumers of OO (>4 times/week) demonstrated lower THR-induced P-selectin expression on the membanes of platelets compared to less frequent consumers (<1 time/week). This study clearly showed that increased consumption of OO correlates with lower platelet activation however the types of OO consumed by the volunteers of the study were not identified [63].

The interventional studies with EVOOs/phenolic extracts in humans are presented in Table 3 (Ref. [45, 64, 65, 66, 67, 68, 69, 70, 71, 72]). One study coming from the PREDIMED trial investigated the effect of a MD regime enriched with either EVOO (MD-EVOO) or nuts (MD-Nuts) on platelet count. Both MD interventions were able to keep platelet counts within the normal range taking into account the trend for an increase of platelet count over time. In addition, both MD interventions decreased the risk of developing thrombocytopenia while blunted the association of thrombocytopenia with all-cause mortality. However, since the beneficial alterations of platelet counts were observed with both MD interventions, they can’t be attributed to EVOO phenolics [64]. Another study from a small subcohort of the PREDIMED [65] compared the effect of interventions on the concentration of plasma microvesicles (MVs) from different cellular origins. Such MVs have both pro-coagulant [73] and pro-inflammatory actions [74] and they are found in higher concentrations in patients suffering from a major adverse cardiovascular event [75]. The study demonstrated that only the MD-Nut arm was able to reduce platelet derived MVs (PAC-1+/AV+ and CD62P+/AV+) and after one year follow up platelet-derived microparticles were lower in the nuts group after compared with the other two groups. Therefore, it seems that EVOO does not have a role on this mechanism although further studies are required.

**Table 3.** **Dietary interventions with pure phenolic compounds, phenolic extracts and EVOO in humans**.

Reference	Study design	Population	Groups/Intervention/Duration	Platelet indices	Outcomes
Oubiña P, 2001 [66]	Two armed, cross-over with no washout period between intervention trials	Non-obese, postmenopausal women (N = 14)	EVOO group: EVOO containing 74% oleic acid and 108 mg/Kg total polyphenols	ADP-induced ${\mathrm{TxB}}_{2}$ production in PRP	At the end of each feeding period:
			HOSO group: High oleic acid sunflower oil containing 73.5% oleic acid and 25 mg/Kg total polyphenols	24 h urine ${\mathrm{TxB}}_{2}$	ADP-induced ${\mathrm{TxB}}_{2}$ concentration in PRP was lower in the EVOO group (584 ± 356 pg/${\mathrm{10}}^{8}$ plts) compared to HOSO (698 ± 369 pg/${\mathrm{10}}^{8}$ plts)
			EVOO and HOSO represented the 62% of total lipid intake	24 h urine 6-keto-prostaglandin-F1α	No significant differences in 24 h urine ${\mathrm{TxB}}_{2}$, 6-keto-prostaglandin-F1α and their ration between the two diets
			Each feeding period lasted 28 d		
Visioli F, 2005 [67]	Two armed, randomized, cross-over with a run in period before treatments and a washout period between treatments	Mildly dyslipidemic patients (N = 22, 10 females)	ROO group: 40 mL/day of refined olive oil containing 2 mg/L phenolics	Serum ${\mathrm{TxB}}_{2}$	EVOO reduced serum ${\mathrm{TxB}}_{2}$ by 21%. No effect of ROO
			EVOO group: 40 mL/day of extra virgin olive oil containing 166mg/L total hydroxytyrosol (HT + HT esterified in OE)		
			3 wks run in period (40 mL ROO) - 7 weeks first arm (40 mL EVOO/ROO) - 4 wks wash out (40 mL ROO) - 7 weeks second arm (40 mL ROO/EVOO)		
Léger CL, 2005 [68]	Single arm, noncontrolled intervention	Type I diabetic patients (N = 5)	HT-rich phenolic extract from olive mill wastewaters consumed with breakfast for four consecutive days (1st day 25 mg HT, the following 3 days 12.5 mg)	Serum ${\mathrm{TxB}}_{2}$ after 30-min clotting	Significant decrease in the ${\mathrm{TxB}}_{2}$ release at day 4 as compared to day 0 (–46.8 ± 10.9%)
			The extract contained 53% HT, 13% TYR in the free form and 34% in elenolic and elenolic acid derivatives		
Widmer RJ, 2013 [72]	Double-blind, controlled, parallel, randomized trial	Patients with early atherosclerosis assessed by a reactive hyperemia-peripheral arterial tonometry (N = 52)	EVOO group: 30 mL/day of EVOO (total polyphenols: 340 mg/Kg) (N = 28)	Platelet count	Reduction of platelet count after supplementation in the combined EVOO groups (Baseline: 242 × ${\mathrm{10}}^{9}$/L, 4 mo: 229 × ${\mathrm{10}}^{9}$/L)
			EVOO + EGCG: 30 mL/day of EVOO containing 280 mg/L EGCG (total polyphenols: 600 mg/Kg) (N = 24)		No difference between groups
			4 months		
Carnevale R, 2014 [70]	Crossover, two armed, postprandial studies	Healthy subjects (N = 25)	**Study 1**	Platelet sNOX2-dp release	**Study 1**
			Phase 1: Mediterranean lunch	Platelet 8-iso-PGF2α-III production	The Mediterranean lunch increased platelet ROS production (27%), platelet sNOX2-dp release (26%) and platelet 8-iso-PGF2α-III production (45%)
			Phase 2: Mediterranean lunch + 10 g EVOO	Platelet ROS production by flow cytometry	The inclusion of 10g EVOO to the lunch almost completely blunted the increases of platelet ROS, sNOX2-dp and 8-iso-PGF2α-III
			**Study 2**		**Study 2**
			Phase 1: Mediterranean lunch + 10 g Corn Oil (CO)		The Mediterranean lunch + CO increased platelet ROS production (38%), platelet sNOX2-dp release (48%) and platelet 8-iso-PGF2α-III production (34%)
			Phase 2: Mediterranean lunch + 10 g EVOO		In the Mediterranean lunch + EVOO no significant changes 2 h after the meal
			30 d interval between phases		
			Blood sampling before lunch and 2 h after lunch		
Agrawal K, 2017 [71]	Randomized, double blind, placebo controlled, crossover acute study	Healthy subjects (N = 9)	40 mL EVOO tyrosol-poor with 1:2 oleacein/oleocanthal ratio	COL-induced maximum platelet aggregation in WB	Ibuprofen treatment reduced COL (3 µg/mL) induced platelet aggregation by 57.5 ± 32.9%
			40 mL EVOO tyrosol-poor with 2:1 oleacein/oleocanthal ratio	COL-induced oxylipin production in PRP	EVOO with 1:2 oleacein/oleocanthal ratio reduced COL (1 µg/mL) induced platelet aggregation by −35 ± 39%
			40 mL EVOO predominantly tyrosol		EVOO with 2:1 oleacein/oleocanthal ratio reduced COL (1 µg/mL) induced platelet aggregation by −13 ± 36%
			400 mg ibuprofen		Regression analyses showed that the oleocanthal provided was the strongest individual ΔPmax predictor (R = 0.563, *p* = 0.002)
			Blood sampling before and 2 hours after EVOO/ibuprofen consumption		Ibuprofen treatment decreased 1 µg/mL COL stimulated oxylipin concentrations
					EVOO intake did not change the 1 µg/mL COL-stimulated oxylipin production
Carnevale R, 2018 [45]	Randomized, double blind, placebo controlled, crossover postprandial study	Healthy subjects (N = 20)	Phase 1: Mediterranean lunch + placebo 20 mg	Platelet 8-iso-PGF2α-III production	A significant difference between the treatments was found for platelet 8-iso-PGF2 and p47phox phosphorylation
			Phase 2: Mediterranean lunch + 20 mg OE	Platelet p47phox phosphoryaltion	Placebo-treated subjects showed increases of 8-iso- PGF2 (45%) and platelet p47phox phosphorylation (212%) 2 h after the meal
			Blood sampling before lunch and 2 h after lunch		OE treated subjects showed a lower increase of
					8-iso-PGF2 (8%) and platelet p47phox phosphorylation (42%)
Chiva-Blanch G, 2020 [65]	Multicentered, randomized, controlled trial	Subcohort of the PREDIMED study (older population at high CVD risk, N = 155)	Control: Advice on low-fat diet (N = 53)	Plasma platelet derived MVs (CD61, PAC-1 and CD62P positive MVs)	MD-Nuts significantly decreased mean platelet-derived cMV
			MD-EVOO: MD enriched with EVOO (N = 53)		Platelet-derived MVs concentrations were lower in the MD-Nuts group after one-year intervention compared with the LFD and EVOO interventions
			MD-Nuts: MD enriched with nuts (N = 49)		
			1 year follow up		
Rus A, 2020 [69]	Randomized, controlled, double-blind, 2-arm parallel study	Female patients diagnosed with fibromyalgia (according to the criteria of the American College of Rheumatology) (N = 30)	EVOO group: 50 mL/day of EVOO (248 mg/Kg total polyphenols)	Platelet count	No significant effect of EVOO on measured parameters
			ROO group: 50 mL/day of ROO (152 mg/Kg total polyphenols)	MPV	ROO increased MPV (Pre: 7.55 ± 0.46 fL, Post: 8.65 ± 1.02 fL) and lowered PDW (Pre: 59.9 ± 11.3%, Post: 48.4 ± 10.1%)
			2wks run in period (50 mL/day ROO) - 3wks intervention (50 mL/day EVOO or ROO)	PDW	Significant time x group effect for PDW (*p* = 0.035)
Hernáez A, 2021 [64]	Multicentered, randomized, controlled trial	Subcohort of the PREDIMED study (older population at high CVD risk, N = 3086)	Control: Advice on low-fat diet (N = 988)	Platelet count	Platelet count
			MD-EVOO: MD enriched with EVOO (N = 1128)		increased over time (+0.98 × ${\mathrm{10}}^{9}$ units/L/year) in the whole population
			MD-Nuts: MD enriched with nuts (N = 970)		Both MD interventions restrained the increase of platelet count in individuals with near-high baseline counts [Time x Group effect, ${\mathrm{10}}^{9}$ units/L/year 95% CI vs Control Diet, MD-EVOO: –2.48 (–5.36; 0.40), MD-Nuts: –4.13 (–7.17–1.09)]
			5 years follow up		
					MD interventions were associated with a decreased risk of developing thrombocytopenia [HR, 95% CI for MD-EVOO: 0.36 (0.16; 0.80), for MD-Nuts: 0.56 (0.26–1.21)]
					Thrombocytopenia was associated with a higher risk of all-cause mortality [HR: 4.71 (2.69; 8.24)]. This association is stronger in the control diet and blunted in the MD groups

ADP, Adenosine diphosphate; CO, Corn Oil; COL, Collagen; CVD, Cardiovascular Disease; MD, Mediterranean diet; EGCG, epigallocatechin gallate; EVOO, Extra virgin olive oil; HOSO, high-oleic acid sunflower oil; HT, Hydroxytyrosol; MVs, microvesicles; LFD, low fat diet; MD, Mediterranean diet; MPV, Mean Platelet Volume; sNOX2-dp, soluble NOX2–derived peptide; OE, oleuropein; PDW, Platelet distribution width; PGF, prostaglandin; PRP, Platelet rich plasma; ROO, Refined Olive Oil; ROS, reactive oxygen species; TYR, Tyrosol; ${\mathrm{TxB}}_{2}$, Thromboxane B2.

However, valid conclusions on the direct effect of OO phenolics to platelet functionality can only be drawn by studies consisting of intervention groups differing in the amount of the administered phenolics and keeping FA profile similar. A small cross-over trial from Oubiña *et al*. [66] supplemented post-menopausal women with either EVOO (total polyphenols: 108 mg/Kg) or high-oleic acid sunflower oil (HOSO) (total polyphenols: 25 mg/Kg). The two oils had similar oleic acid content but HOSO contained higher concentrations of α-tcocpherol, total sterols and much lower concentration of squalene. Despite the modest concentration of total polyphenols in the EVOO, its administration lowered ${\mathrm{TxB}}_{2}$ production in PRP induced by ADP compared to HOSO. However, no differences were observed in the concentration of ${\mathrm{TxB}}_{2}$ and 6-keto-prostaglandin-F1α (a stable analogue of prostacyclin in 24 h urine samples). The authors attributed this difference to the higher amount of linoleic acid in the HOSO diet, however according to the aforementioned cell and animal studies, the increased content of polyphenols in EVOO may play a role for the observed antiplatelet effect of EVOO [66]. Stronger evidence for the ability of EVOO to attenuate chronic platelet activation was obtained by the cross-over VOLOS study. This study demonstrated the ability of EVOO, at doses (40 mL/day), comparable to those consumed by the Mediterranean populations to reduce serum ${\mathrm{TxB}}_{2}$ production by 20%. EVOO also improved plasma antioxidant capacity raising the possibility that the redox modulation of EVOO polyphenols may play a role on EVOOs antiplatelet properties [67]. The outcomes of VOLOS were confirmed by a very small, single-armed study in Type 1 diabetic patients who are administered with a HT-rich phenolic extract from olive mill wastewaters for 4 consecutive days corresponding to the amount of HT typically found in 25–50 g of olive fruit. A significant reduction (47%) of serum ${\mathrm{TxB}}_{2}$ after 30-min clotting was observed at the fourth day indicating the ability of dietary HT to attenuate platelet aggregability *in vivo * [68]. Another study in female patients with fibromyalgia compared an EVOO (248 mg/Kg total polyphenols) with an ROO of similar fatty acid profile but poorer in polyphenols (152 mg/Kg total polyphenols) to affect cardiovascular risk factors [69]. The EVOO did not have an effect of platelet count, mean platelet volume (MPV) and platelet distribution width (PDW) while ROO lowered PDW compared to EVOO. These results are in contrast with the aforementioned PREDIMED study where MD-EVOO could modulate platelet counts [64], however we have to mention that this study was much smaller, the intervention lasted only 3weeks and the difference in the total polyphenol content between EVOO and ROO was rather small.

The postprandial state is a daily, metabolically challenging period for the human metabolism and several homeostatic mechanisms are activated for the proper handling of macronutrients. Energy dense meals can stress those mechanisms leading to a transient, postprandial hyperglycemia, hyperlipidemia often accompanied by proinflammatory processes and oxidative stress [76]. Taking into account that OO is usually consumed as part of a meal and that postprandial dysmetabolism is a risk factor for atherogenesis, the ability of EVOO to handle postprandial oxidative stress may give nutritional relevant explanations for its cardioprotective properties. Under this perspective, Carnevale *et al*. [70] conducted two postprandial studies comparing the effect of a typical Mediterranean lunch with or without corn oil (CO) and EVOO on platelet ROS production, platelet lipid peroxidation and activation of NOX. The studies showed that EVOO almost completely blunted the postprandial increases of these oxidative stress indices. *In vitro* studies have shown that the postprandial antioxidant effects of EVOO can be attributed to both its vitamin E and polyphenol content [70]. A similar follow up study was conducted a few years later from the same group. Just before a typical lunch healthy volunteers received either placebo or 20 mg OE, a precursor of HT in OO. The outcomes of the study clearly showed that OE could minimize postprandial lipid peroxidation in platelets and p47phox phosphorylation. The latter is the cytosolic subunit of platelets’ Nox2 and its phosphorylation leads to Nox2 activation. The *in vivo* results were confirmed by *in vitro* data showing that HT reduced p47phox phosphorylation and isoprostane formation [45]. The results of the aforementioned studies indicate that platelets are an important source of ROS postprandially and a target for the antioxidant phenols of EVOO. Moreover, the downregulation of Nox2-derived oxidative stress can improve hyperglycemia by increasing the bioavailabilty of incretins as the authors showed in the same study [45]. Finally, a study of Agrawal K *et al*. [71] comparing the acute effect of 40 mL EVOO rich either in oleocanthal/oleacein or TYR has shown that oleocanthal/oleacein rich EVOOs can acutely (in 2 h) reduce COL-induced platelet activation without affecting oxylipin production by platelets. The inhibition of platelet aggregation correlated with the oleocanthal content while the inhibition of eicosanoid production with the EVOO total phenolic content. Oleocanthal is strong inhibitor of COX acting similarly to aspirin in inhibiting platelet activation [71].

A relatively recent research trend in the field of nutrition is the enrichment of foods with bioactive extracts or pure compounds in an attempt to formulate food products with pleiotropic bioactions. Under this perspective the group of Widmer *et al*. [72] produced an EVOO enriched with green tea catechins (EGCG) and compared it with the non-enriched EVOO in early-atherosclerotic patients. Among other parameters, they measured platelet count and they found that in the combined EVOO groups OO supplementation could lower platelet count at the end of the supplementation period with no differences between groups. Subclinical inflammation and cytokines such as interleukin 6 (IL-6) are potent thrombopoietic factors [77] so the observed reduction in platelet counts could be attributed to an improvement of subclinical inflammation. Although white blood cells (WBC) counts and plasma soluble intercellular adhesion molecule-1 (sICAM) were reduced by both EVOOs other inflammatory markers were not affected significantly implying that other mechanisms could be involved in the modulation of platelet counts after the consumption of EVOO [72].

Conclusively, the data from human studies are promising but again not persuasive mainly because the majority of studies are under-powered and heterogenous concerning the type of the supplemented EVOOs/extracts, the dosage schemes, the subjects and the assays of platelet functionality. We clearly need more large cohort studies, like the ones coming from the PREDIMED trial, where the modulation of platelet indices by OO phenolics should be linked to hard end-points. If so, then the notion that the antiplatelet properties of OO phenolics may be partly responsible for their cardioprotective properties will be strengthened.

## 8. Conclusions 

A plethora of studies so far clearly demonstrate that the hyperactivity of platelets, especially under insulin resistance conditions (obesity, metabolic syndrome, diabetes), can augment the pathogenesis of several non-communicable disease including CVDs, cancer and neurological disorders. At the same time recent, large, cohort studies and meta-analyses have shown that the MD and its main component, EVOO, are able to beneficially modulate the progression of those disease. It is also more than clear, that the phenolic content of OO (both its quantity and its composition) is responsible, either alone or in combination with the other micrconstituents of OO for its biological properties. Therefore, the notion that OO phenolics have antiplatelet actions and this is an important mechanism by which EVOO exert its health protective actions is justified. The cellular and animal studies clearly show that EVOO phenolics, mainly HT, are able to inhibit the activation of platelets induced by several endogenous agonists and pathologies. They also showed that OO by-products are a promising source of phenolic extracts which can be utilized in the development of supplements and nutraceuticals with promising antiplatelet actions. In addition, they demonstrated that small chemical modifications of OO phenolics towards increased lipophilicity may lead to the development of derivatives with augmented bioavailability and antiplatelet actions. However, the outcomes of the pre-clinical studies are difficult to be translated to humans mainly because the dosages and the chemical forms of the phenolics used to these studies are much higher and different to that found in human circulation. Despite the heterogeneity of the few human trials on the field so far, the results are promising showing that EVOO can exert antiplatelet actions in real, acute or long-term, trials and at least part of this antiplatelet action can be attributed to the phenolic content of EVOOs. However, we clearly need better, well-powered studies to give answers on the ideal composition of phenolics in EVOO, the daily doses that they should be administered according to the pathology of the population and mechanistic issues concerning the bioavailability and the mode of action on platelets. Last but not least the antiplatelet properties of OO phenolics should be correlated with hard end-points. In any case, the antiplatelet properties of OO phenolics is a promising, emerging mechanism which may explain some of the health properties of EVOO and the MD.

## Abbreviations

Aβ, Amyloid beta peptide; AA, Arachidonic Acid; AD, Alzheimer’s disease; ADP, Adenosine diphosphate; AP, Apigenin; ASA, Acetylsalicylic acid; BW, Body Weight; cAMP, cyclic 3’ 5’ adenosine monophosphate; cGMP, Cyclic guanosine monophosphate; CO, corn oil; COL, Collagen; COX, cyclooxygenase; CVD, Cardiovascular Disease; DACOLAG, Deacetoxy oleuropein aglycone; DHPG, 3,4-dihydroxyphenylglycol; DR, diabetic rats; EGCG, enriched with green tea catechins; EVOO, Extra virgin olive oil; FA, Fatty acids; GAE, Gallic acid equivalents; HC, high MPC concentration; 12-HETE, 12-hydroxy-5,8,10,14-eicosatetraenoic acid; HHT, 12-hydroxy-5,8,10-heptadecatrienoic acid; HOSO, High-oleic acid sunflower oil; HSP, Heat Shock Protein; HT, Hydroxytyrosol; HTA, Hydroxytyrosol acetate; HVA, Homovanillic alcohol; ${\mathrm{ID}}_{50}$, Inhibitory dose for 50% inhibiton; IL-6, interleukin 6; LC, low MPC concentration; LDL, low density lipoprotein; LFD, low fat diet; LU, Luteolin; MAO, Monoamine oxidase; MD, Mediterranean Diet; MI, myocardial infarction; MPC, Minor Polar Compounds; MPV, Mean Platelet Volume; MUFA, Monounsaturated fatty acids; MVs, Microvesicles; NADPH, Nicotinamide adenine dinucleotide phosphate; NDR, Nondiabetic rats; NETs, Neutrophil Extracellular Traps; NLD, normolipemic diet; NO, nitric oxide; NOX, NADPH oxidase; OE, Oleuropein; OEA, Oleuropein aglycon; OO, Olive Oil; oxLDL, oxidized LDL; PAF, Platelet-activating factor; PDW, Platelet distribution width; PDE, phosphodiesterase; PDGF, Platelet-Derived Growth Factor; PE, Phenolic extracts; PGF, prostaglandin; PLT, platelet; PRP, Platelet rich plasma; PPF, Polymeric phenolic fractions; PUFA, Polyunsaturated fatty acids; rHcy, reduced homocysteine; ROS, reactive oxygen species; ROO, Refined Olive Oil; QU, flavonoids quercetin; SA, Sodium Arachidonate; sICAM, Plasma soluble intercellular adhesion molecule; sCD40L, Soluble CD40L; sNOX2-dp, soluble NOX2–derived peptide; SFA, saturated fatty acids; SFAED, saturated fatty acid-enriched diet; TPL, Total Phosholipids; T2DM, Type 2 Diabetes Mellitus; THR, Thrombin; TRAP, Thrombin Receptor Activating Peptide; ${\mathrm{TxA}}_{2}$, Thromboxane A2; ${\mathrm{TxB}}_{2}$, Thromboxane B2; TYR, Tyrosol; WB, Whole Blood; WBC, white blood cells; WW, Waste Water.

## References

[1] Dinu M, Pagliai G, Casini A, Sofi F (2018). Mediterranean diet and multiple health outcomes: an umbrella review of meta-analyses of observational studies and randomised trials. *European Journal of Clinical Nutrition*.

[2] Guasch‐Ferré M, Willett WC (2021). The Mediterranean diet and health: a comprehensive overview. *Journal of Internal Medicine*.

[3] Trichopoulou A, Martínez-González MA, Tong TY, Forouhi NG, Khandelwal S, Prabhakaran D (2014). Definitions and potential health benefits of the Mediterranean diet: views from experts around the world. *BMC Medicine*.

[4] Gaforio JJ, Visioli F, Alarcón-de-la-Lastra C, Castañer O, Delgado-Rodríguez M, Fitó M (2019). Virgin Olive Oil and Health: Summary of the III International Conference on Virgin Olive Oil and Health Consensus Report, JAEN (Spain) 2018. *Nutrients*.

[5] Gorzynik-Debicka M, Przychodzen P, Cappello F, Kuban-Jankowska A, Marino Gammazza A, Knap N (2018). Potential Health Benefits of Olive Oil and Plant Polyphenols. *International Journal of Molecular Sciences*.

[6] Gresele P, Lopez JA, Kleiman NS, Page CP (2017). *Platelets in thrombotic and non-thrombotic disorders: Pathophysiology, pharmacology and therapeutics: An update*.

[7] Zamora C, Cantó E, Vidal S (2021). The Dual Role of Platelets in the Cardiovascular Risk of Chronic Inflammation. *Frontiers in Immunology*.

[8] Lisman T, Luyendyk J (2018). Platelets as Modulators of Liver Diseases. *Seminars in Thrombosis and Hemostasis*.

[9] Camilli M, Iannaccone G, La Vecchia G, Cappannoli L, Scacciavillani R, Minotti G (2021). Platelets: the point of interconnection among cancer, inflammation and cardiovascular diseases. *Expert Review of Hematology*.

[10] Deppermann C (2018). Platelets and vascular integrity. *Platelets*.

[11] Tsoumani ME, Tselepis AD (2017). Antiplatelet Agents and Anticoagulants: from Pharmacology to Clinical Practice. *Current Pharmaceutical Design*.

[12] Jourdi G, Lordkipanidzé M, Philippe A, Bachelot-Loza C, Gaussem P (2021). Current and Novel Antiplatelet Therapies for the Treatment of Cardiovascular Diseases. *International Journal of Molecular Sciences*.

[13] McEwen B (2014). The Influence of Diet and Nutrients on Platelet Function. *Seminars in T hrombosis and Hemostasis*.

[14] Bachmair EM, Ostertag LM, Zhang X, de Roos B (2014). Dietary manipulation of platelet function. *Pharmacology and Therapeutics*.

[15] Gremmel T, Frelinger AL, Michelson AD (2016). Platelet Physiology. *Seminars in Thrombosis and Hemostasis*.

[16] Goto S, Hasebe T, Takagi S (2015). Platelets: Small in Size but Essential in the Regulation of Vascular Homeostasis - Translation from Basic Science to Clinical Medicine. *Circulation Journal*.

[17] Nording H, Baron L, Langer HF (2020). Platelets as therapeutic targets to prevent atherosclerosis. *Atherosclerosis*.

[18] Baigent C, Blackwell L, Collins R, Emberson J, Godwin J, Peto R (2009). Aspirin in the primary and secondary prevention of vascular disease: collaborative meta-analysis of individual participant data from randomised trials. *The Lancet*.

[19] Bowman L, Mafham M, Wallendszus K, Stevens W, Buck G, Barton J (2018). Effects of Aspirin for Primary Prevention in Persons with Diabetes Mellitus. *The New England Journal of Medicine*.

[20] McNeil JJ, Wolfe R, Woods RL, Tonkin AM, Donnan GA, Nelson MR (2018). Effect of Aspirin on Cardiovascular Events and Bleeding in the Healthy Elderly. *New England Journal of Medicine*.

[21] Flossmann E, Rothwell PM (2007). Effect of aspirin on long-term risk of colorectal cancer: consistent evidence from randomised and observational studies. *The Lancet*.

[22] Clarke CA, Canchola AJ, Moy LM, Neuhausen SL, Chung NT, Lacey JV (2017). Regular and low-dose aspirin, other non-steroidal anti-inflammatory medications and prospective risk of HER2-defined breast cancer: the California Teachers Study. *Breast Cancer Research*.

[23] Vidal AC, Howard LE, Moreira DM, Castro-Santamaria R, Andriole GL, Freedland SJ (2015). Aspirin, NSAIDs, and Risk of Prostate Cancer: Results from the REDUCE Study. *Clinical Cancer Research*.

[24] McNeil JJ, Gibbs P, Orchard SG, Lockery JE, Bernstein WB, Cao Y (2021). Effect of Aspirin on Cancer Incidence and Mortality in Older Adults. *Journal of the National Cancer Institute*.

[25] Mauri L, Kereiakes DJ, Yeh RW, Driscoll-Shempp P, Cutlip DE, Steg PG (2014). Twelve or 30 Months of Dual Antiplatelet Therapy after Drug-Eluting Stents. *New England Journal of Medicine*.

[26] Kuan YC, Huang KW, Lin CL, Luo JC, Kao CH (2019). Effects of Aspirin or Clopidogrel on Colorectal Cancer Chemoprevention in Patients with Type 2 Diabetes Mellitus. *Cancers*.

[27] Stellos K, Panagiota V, Kögel A, Leyhe T, Gawaz M, Laske C (2010). Predictive Value of Platelet Activation for the Rate of Cognitive Decline in Alzheimer’s Disease Patients. *Journal of Cerebral Blood Flow and Metabolism*.

[28] Stellos K, Katsiki N, Tatsidou P, Bigalke B, Laske C (2014). Association of Platelet Activation with Vascular Cognitive Impairment: Implications in Dementia Development. *Current Vascular Pharmacology*.

[29] Randriamboavonjy V, Sopova K, Stellos K, Laske C (2014). Platelets as potential link between diabetes and Alzheimer’s disease. *Current Alzheimer Research*.

[30] Rollini F, Franchi F, Muñiz-Lozano A, Angiolillo DJ (2013). Platelet Function Profiles in Patients with Diabetes Mellitus. *Journal of Cardiovascular Translational Research*.

[31] Kaur R, Kaur M, Singh J (2018). Endothelial dysfunction and platelet hyperactivity in type 2 diabetes mellitus: molecular insights and therapeutic strategies. *Cardiovascular Diabetology*.

[32] Souza PAL, Marcadenti A, Portal VL (2017). Effects of Olive Oil Phenolic Compounds on Inflammation in the Prevention and Treatment of Coronary Artery Disease. *Nutrients*.

[33] Jimenez-Lopez C, Carpena M, Lourenço-Lopes C, Gallardo-Gomez M, Lorenzo JM, Barba FJ (2020). Bioactive Compounds and Quality of Extra Virgin Olive Oil. *Foods*.

[34] Rigacci S, Stefani M (2016). Nutraceutical Properties of Olive Oil Polyphenols. An Itinerary from Cultured Cells through Animal Models to Humans. *International Journal of Molecular Sciences*.

[35] Grosso G, Marventano S, Yang J, Micek A, Pajak A, Scalfi L (2017). A comprehensive meta-analysis on evidence of Mediterranean diet and cardiovascular disease: are individual components equal. *Critical Reviews in Food Science and Nutrition*.

[36] Estruch R, Ros E, Salas-Salvadó J, Covas MI, Corella D, Arós F (2018). Primary Prevention of Cardiovascular Disease with a Mediterranean Diet Supplemented with Extra-Virgin Olive Oil or Nuts. *The New England Journal of Medicine*.

[37] Schwingshackl L, Hoffmann G (2014). Monounsaturated fatty acids, olive oil and health status: a systematic review and meta-analysis of cohort studies. *Lipids in Health and Disease*.

[38] Zhu Y, Bo Y, Liu Y (2019). Dietary total fat, fatty acids intake, and risk of cardiovascular disease: a dose-response meta-analysis of cohort studies. *Lipids in Health and Disease*.

[39] Karković Marković A, Torić J, Barbarić M, Jakobušić Brala C (2019). Hydroxytyrosol, Tyrosol and Derivatives and Their Potential Effects on Human Health. *Molecules*.

[40] Martucci M, Ostan R, Biondi F, Bellavista E, Fabbri C, Bertarelli C (2017). Mediterranean diet and inflammaging within the hormesis paradigm. *Nutrition Reviews*.

[41] Togna GI, Togna AR, Franconi M, Marra C, Guiso M (2003). Olive Oil Isochromans Inhibit Human Platelet Reactivity. *The Journal of Nutrition*.

[42] Turner R, Etienne N, Garcia Alonso M, de Pascual-Teresa S, Minihane AM, Weinberg PD (2005). Antioxidant and Anti-atherogenic Activities of Olive Oil Phenolics. *International Journal for Vitamin and Nutrition Research*.

[43] Pathania A, Kumar R, Sandhir R (2021). Hydroxytyrosol as anti-parkinsonian molecule: Assessment using in-silico and MPTP-induced Parkinson’s disease model. *Biomedicine and Pharmacotherapy*.

[44] Fragopoulou E, Nomikos T, Karantonis HC, Apostolakis C, Pliakis E, Samiotaki M (2007). Biological Activity of Acetylated Phenolic Compounds. *Journal of Agricultural and Food Chemistry*.

[45] Carnevale R, Nocella C, Cammisotto V, Bartimoccia S, Monticolo R, D’Amico A (2018). Antioxidant activity from extra virgin olive oil via inhibition of hydrogen peroxide–mediated NADPH-oxidase 2 activation. *Nutrition*.

[46] Petroni A, Blasevich M, Salami M, Papini N, Montedoro GF, Galli C (1995). Inhibition of platelet aggregation and eicosanoid production by phenolic components of olive oil. *Thrombosis Research*.

[47] de Roos B, Zhang X, Rodriguez Gutierrez G, Wood S, Rucklidge GJ, Reid MD (2011). Anti-platelet effects of olive oil extract: in vitro functional and proteomic studies. *European Journal of Nutrition*.

[48] Mizutani D, Onuma T, Tanabe K, Kojima A, Uematsu K, Nakashima D (2020). Olive polyphenol reduces the collagen-elicited release of phosphorylated HSP27 from human platelets. *Bioscience, Biotechnology, and Biochemistry*.

[49] Dell’Agli M, Maschi O, Galli GV, Fagnani R, Dal Cero E, Caruso D (2008). Inhibition of platelet aggregation by olive oil phenols via cAMP-phosphodiesterase. *British Journal of Nutrition*.

[50] Correa JA, López-Villodres JA, Asensi R, Espartero JL, Rodríguez-Gutiérez G, De La Cruz JP (2009). Virgin olive oil polyphenol hydroxytyrosol acetate inhibits in vitro platelet aggregation in human whole blood: comparison with hydroxytyrosol and acetylsalicylic acid. *The British Journal of Nutrition*.

[51] Reyes JJ, De La Cruz JP, Muñoz-Marin J, Guerrero A, Lopez-Villodres JA, Madrona A (2013). Antiplatelet effect of new lipophilic hydroxytyrosol alkyl ether derivatives in human blood. *European Journal of Nutrition*.

[52] Zbidi H, Salido S, Altarejos J, Perez-Bonilla M, Bartegi A, Rosado JA (2009). Olive tree wood phenolic compounds with human platelet antiaggregant properties. *Blood Cells, Molecules, and Diseases*.

[53] Rubio-Senent F, de Roos B, Duthie G, Fernández-Bolaños J, Rodríguez-Gutiérrez G (2015). Inhibitory and synergistic effects of natural olive phenols on human platelet aggregation and lipid peroxidation of microsomes from vitamin E-deficient rats. *European Journal of Nutrition*.

[54] Antonopoulou S, Detopoulou M, Fragopoulou E, Nomikos T, Mikellidi A, Yannakoulia M (2022). Consumption of yogurt enriched with polar lipids from olive oil by-products reduces platelet sensitivity against platelet activating factor and inflammatory indices: a randomized, double-blind clinical trial. *Human Nutrition and Metabolism*.

[55] De La Cruz JP, Villalobos MA, Carmona JA, Martín-Romero M, Smith-Agreda JM, de la Cuesta FS (2000). Antithrombotic Potential of Olive Oil Administration in Rabbits with Elevated Cholesterol. *Thrombosis Research*.

[56] De La Cruz JP, Del Río S, López-Villodres JA, Villalobos MA, Jebrouni N, González-Correa JA (2010). Virgin olive oil administration improves the effect of aspirin on retinal vascular pattern in experimental diabetes mellitus. *British Journal of Nutrition*.

[57] De La Cruz Cortés JP, Vallejo-Carmona L, Arrebola MM, Martín-Aurioles E, Rodriguez-Pérez MD, Ortega-Hombrados L (2021). Synergistic Effect of 3’,4’-Dihidroxifenilglicol and Hydroxytyrosol on Oxidative and Nitrosative Stress and Some Cardiovascular Biomarkers in an Experimental Model of Type 1 Diabetes Mellitus. *Antioxidants*.

[58] González-Correa JA, Muñoz-Marín J, Arrebola MM, Guerrero A, Narbona F, López-Villodres JA (2007). Dietary Virgin Olive Oil Reduces Oxidative Stress and Cellular Damage in Rat Brain Slices Subjected to Hypoxia-Reoxygenation. *Lipids*.

[59] Priora R, Summa D, Frosali S, Margaritis A, Di Giuseppe D, Lapucci C (2008). Administration of Minor Polar Compound-Enriched Extra Virgin Olive Oil Decreases Platelet Aggregation and the Plasma Concentration of Reduced Homocysteine in Rats. *The Journal of Nutrition*.

[60] González-Correa JA, Navas MD, Muñoz-Marín J, Trujillo M, Fernández-Bolaños J, de la Cruz JP (2008). Effects of Hydroxytyrosol and Hydroxytyrosol Acetate Administration to Rats on Platelet Function Compared to Acetylsalicylic Acid. *Journal of Agricultural and Food Chemistry*.

[61] Muñoz-Marín J, De La Cruz JP, Reyes JJ, López-Villodres JA, Guerrero A, López-Leiva I (2013). Hydroxytyrosyl alkyl ether derivatives inhibit platelet activation after oral administration to rats. *Food and Chemical Toxicology*.

[62] Coppola A, Davi G, De Stefano V, Mancini FP, Cerbone AM, Di Minno G (2000). Homocysteine, Coagulation, Platelet Function, and Thrombosis. *Seminars in Thrombosis and Hemostasis*.

[63] Zhang R, Moscona A, Myndzar K, Luttrell-Williams E, Vanegas S, Jay MR (2021). More frequent olive oil intake is associated with reduced platelet activation in obesity. *Nutrition, Metabolism and Cardiovascular Diseases*.

[64] Hernáez Á, Lassale C, Castro-Barquero S, Ros E, Tresserra-Rimbau A, Castañer O (2021). Mediterranean Diet Maintained Platelet Count within a Healthy Range and Decreased Thrombocytopenia-Related Mortality Risk: A Randomized Controlled Trial. *Nutrients*.

[65] Chiva-Blanch G, Sala-Vila A, Crespo J, Ros E, Estruch R, Badimon L (2020). The Mediterranean diet decreases prothrombotic microvesicle release in asymptomatic individuals at high cardiovascular risk. *Clinical Nutrition*.

[66] Oubiña P, Sánchez-Muniz FJ, Ródenas S, Cuesta C (2001). Eicosanoid production, thrombogenic ratio, and serum and LDL peroxides in normo- and hypercholesterolaemic post-menopausal women consuming two oleic acid-rich diets with different content of minor components. *British Journal of Nutrition*.

[67] Visioli F, Caruso D, Grande S, Bosisio R, Villa M, Galli G (2005). Virgin Olive Oil Study (VOLOS): vasoprotective potential of extra virgin olive oil in mildly dyslipidemic patients. *European Journal of Nutrition*.

[68] Léger CL, Carbonneau MA, Michel F, Mas E, Monnier L, Cristol JP (2005). A thromboxane effect of a hydroxytyrosol-rich olive oil wastewater extract in patients with uncomplicated type I diabetes. *European Journal of Clinical Nutrition*.

[69] Rus A, Molina F, Martínez-Ramírez MJ, Aguilar-Ferrándiz ME, Carmona R, Del Moral ML (2020). Effects of Olive Oil Consumption on Cardiovascular Risk Factors in Patients with Fibromyalgia. *Nutrients*.

[70] Carnevale R, Pignatelli P, Nocella C, Loffredo L, Pastori D, Vicario T (2014). Extra virgin olive oil blunt post-prandial oxidative stress via NOX2 down-regulation. *Atherosclerosis*.

[71] Agrawal K, Melliou E, Li X, Pedersen TL, Wang SC, Magiatis P (2017). Oleocanthal-rich extra virgin olive oil demonstrates acute anti-platelet effects in healthy men in a randomized trial. *Journal of Functional Foods*.

[72] Widmer RJ, Freund MA, Flammer AJ, Sexton J, Lennon R, Romani A (2013). Beneficial effects of polyphenol-rich olive oil in patients with early atherosclerosis. *European Journal of Nutrition*.

[73] Ridger VC, Boulanger CM, Angelillo-Scherrer A, Badimon L, Blanc-Brude O, Bochaton-Piallat M (2017). Microvesicles in vascular homeostasis and diseases. Position Paper of the European Society of Cardiology (ESC) Working Group on Atherosclerosis and Vascular Biology. *Thrombosis and Haemostasis*.

[74] Cortez-Espinosa N, Perez-Campos Mayoral L, Perez-Campos E, Alejandro Cabrera Fuentes H, Perez-Campos Mayoral E, Martínez-Cruz R (2017). Platelets and Platelet-Derived Microvesicles as Immune Effectors in Type 2 Diabetes. *Current Vascular Pharmacology*.

[75] Chiva-Blanch G, Crespo J, Suades R, Arderiu G, Padro T, Vilahur G (2016). CD142+/CD61+, CD146+ and CD45+ microparticles predict cardiovascular events in high risk patients following a Mediterranean diet supplemented with nuts. *Thrombosis and Haemostasis*.

[76] Sottero B, Gargiulo S, Russo I, Barale C, Poli G, Cavalot F (2015). Postprandial Dysmetabolism and Oxidative Stress in Type 2 Diabetes: Pathogenetic Mechanisms and Therapeutic Strategies. *Medicinal Research Reviews*.

[77] Ishibashi T, Kimura H, Shikama Y, Uchida T, Kariyone S, Hirano T (1989). Interleukin-6 is a potent thrombopoietic factor in vivo in mice. *Blood*.

